# RNAdigest: A Web-Based Tool for the Analysis and Prediction of Structure - Specific RNAse Digestion Results

**DOI:** 10.1371/journal.pone.0096759

**Published:** 2014-05-06

**Authors:** Piotr Madanecki, Susan Nozell, Renata Ochocka, James F. Collawn, Rafal Bartoszewski

**Affiliations:** 1 Department of Biology and Pharmaceutical Botany, Medical University of Gdansk, Gdansk, Poland; 2 Department of Cell, Developmental, and Integrative Biology, University of Alabama at Birmingham, Birmingham, Alabama, United States of America; International Centre for Genetic Engineering and Biotechnology, Italy

## Abstract

Despite recent developments in analyzing RNA secondary structures, relatively few RNA structures have been determined. To date, many investigators have relied on the traditional method of using structure-specific RNAse enzymes to probe RNA secondary structures. However, if these data were combined with novel computational approaches, investigators would have an informative and valuable tool for RNA structural analysis. To this end, we created the web server “RNAdigest.” RNAdigest uses mfold RNA structural models in order to predict the results of RNAse digestion experiments. Furthermore, RNAdigest also utilizes both RNA sequence and the experimental digestion patterns to formulate the constraints for predicting secondary structures of the RNA. Thus, RNAdigest allows for the structural interpretation of RNAse digestion experiments. Overall, RNAdigest simplifies RNAse digestion result analyses while allowing for the identification of unique fragments. These unique fragments can then be used for testing predicted mfold structures and for designing structural-specific DNA/RNA probes.

## Introduction

Recent advances in experimental and computational approaches for predicting and analyzing RNA secondary structure, along with the use of deep sequencing technology, have contributed to the prediction and establishment of accurate structures for many RNAs [1], [2]. Despite these *in silico* improvements, deep sequencing has limited availability, and moreover, it is still necessary to validate those predicted structures experimentally. RNAses have high structural specificities and can be used to digest end-labeled RNA molecules under conditions of limited cleavage in order to determine RNA secondary structures. The results of RNAse digestion are visualized on sequencing gels, and the presence of unique structural motifs related to specific digestion products can be confirmed with PCR and northern blots using well-designed primers or probes. Thus despite its limitations, enzymatic RNA structure probing remains the most widely used approach to explore RNA structure and provide detailed structural information on both small and large RNA molecules [3]. Therefore, there is a need for a freely-available, simple and user-friendly software that facilitates the biochemical validation of theoretical structural models and allows the user to incorporate the derived biochemical data (as constraints) into predictive models.

To date, several bioinformatics approaches have been used to predict digestion patterns based on RNA structural predictions [4]–[6] and to simulate the autoradiograms of gel-separated RNA fragments [7]. Our program offers an advantage over the previous versions because it operates independent hardware and is user-friendly and easily accessible to all investigators. Furthermore, RNAdigest incorporates all the best features of the previous programs and uses the most popular tool for RNA structure prediction, mfold [8] (based on free energy minimization algorithms). **RNAdigest** is a user-friendly web tool for (**1**) identifying structural-specific RNAse restriction sites based on the mfold RNA secondary structure program; (**2**) identifying sequences of unique RNAse digestion products; and most importantly, (**3**) computing the mRNA secondary structure constraints based on experimentally obtained sequences of the digestion products.

## Design and Implementation

### RNAdigest Web Server

The RNAdigest uses a graphic, HTML-based interface. We have used PHP, as the main programming language, while JavaScript was employed for the user side. The dynamic-generated graphics are shown in the Scalable Vector Graphics (SVG) format. This feature requires an SVG-compliant browser (Firefox, Internet Explorer (9+), or Google Chrome).

**Figure 1:**
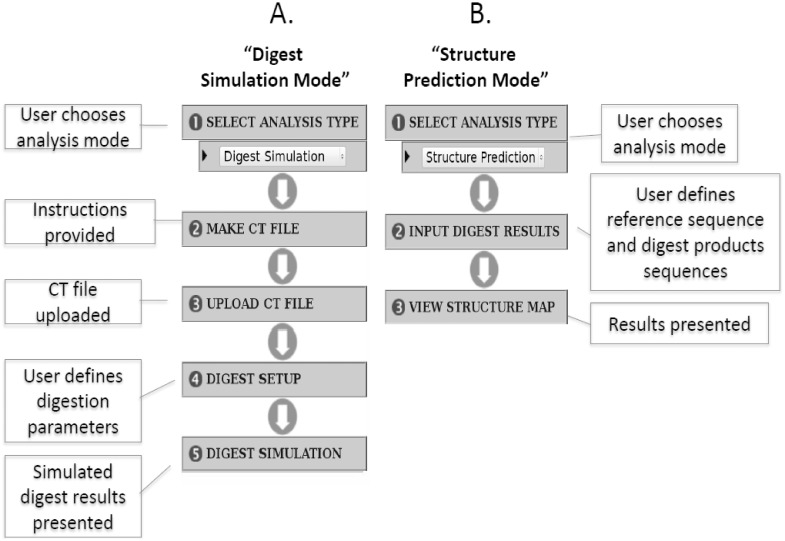
Schematic diagram of typical “step by step” workflows for RNAdigest. **A**. Digest simulation mode is shown. **B**. Structure prediction mode is shown.

The primary goal is to make the software easy to use by presenting the user guide and all linked-tools directly in the main screen. In this respect, the interface construction of RNAdigest is similar to program wizards used by many desktop applications. Initially, the user chooses the work mode and is then guided step-by-step to the results page. The user can choose to go through steps sequentially, or has the ability to jump directly to any selected step in the program. Some of the dynamic-generated graphics are shown in the SVG format. This feature requires an SVG-compliant browser (i.e. Mozilla Firefox,), but offers the advantage of interactivity. For example, when the user selects a digest fragment sequence in the digest product list, the corresponding fragment in both the simulated electrophoresis separation and digestion map are also selected and highlighted. In order to visualize the structural models, the web server integrates sir_graph (from mfold_utility package)[8]. The files generated by sir_graph are in PS (postscript), JPG and PNG formats. RNAdigest operates in two independent modes (*Figure 1*): (1) to predict RNAse digestion results **(“digest simulation” mode)**; and (2) to calculate structure constraints based on experimentally obtained fragments **(“structure prediction” mode)**.

### Input

For the **“digest simulation” mode**, RNAdigest accepts RNA structural models in the form of mfold CT files [8], [9] and Vienna’s dot-bracket notation files [10]. For the **“structure prediction” mode),** RNAdigest accepts sequences in the text and FASTA formats.

**Figure 2:**
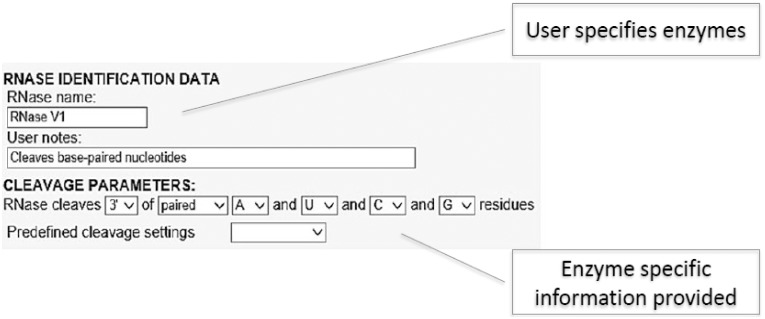
RNAse selection screen for RNAse V1.

In the **“digest simulation” mode**, the user first uploads a RNA structure model file. While uploading this, the software creates a user-assigned directory structure for storing and processing the user’s data. The top directory name in this directory structure is unique and is based on the IP number of that user’s computer. Since all of the data, including user settings are stored in text files within the user directory structure on the RNAdigest server hard disk drive, there is no need to use the database system. Therefore, once the RNAse parameters are established, the RNA digest can be simulated. First, while processing the sequence, the paired/unpaired status of every residue is read from user’s file and the residues that are recognized by the RNAse pairing status are identified. Next, digest simulation results are simulated on an info page that is created and sent to the user’s web browser. This page includes tables with the overall RNAse-specific information and statistics. This information is also simultaneously saved in the user’s directory on the RNAdigest server hard disk drive in the form of downloadable, tab-delimited text files. Graphic files are also generated and saved to the server hard disk drive in SVG, JPG, PS and PNG formats and can be downloaded. RNAdigest uses two methods to generate visualizations: an internal, built-in function and an external program sir_graph (from the mfold_utility package). The graphics files in SVG format are generated by a built-in function. The graphics file, which visualizes structures in JPG, PNG and PS formats, is generated by the sir_graph program using dynamically-generated files to color code residues (recognized and unrecognized residues are assigned in different colors). Furthermore, RNAdigest allows users to define their own cleavage parameters for any structural-specific RNAse that is based on recognized residues and pairing status (*Figure 2*).

**Figure 3:**
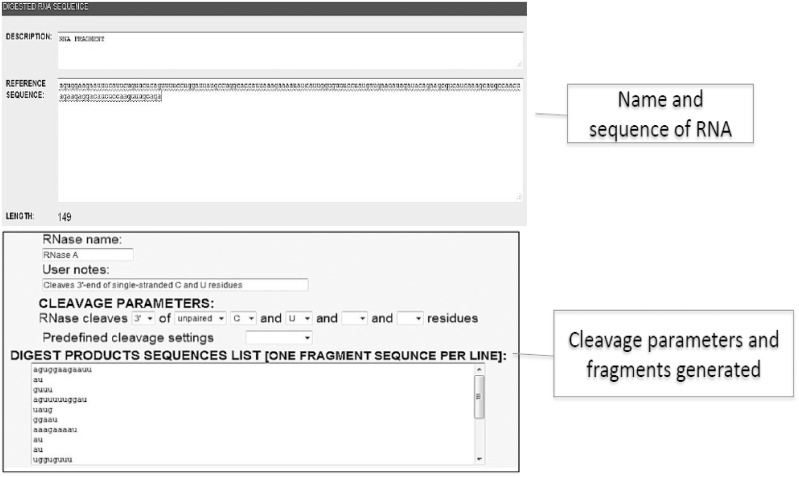
“Structure prediction” mode dynamic form.

In the alternative **“structure prediction” mode**, the user inputs the digestion results: a reference digested RNA sequence, RNAse cleavage parameters, and the produced fragment sequences as shown in *Figure 3*. Data can be typed or pasted into the setup form. Because this information is saved as files on the server hard disk drive when a user submits these forms, the user does not have to re-enter the same information again. During this initial step, the compatibility between the original sequence and digestion products is validated. Each RNA digest product sequence occurrence is identified in the original RNA sequence. The array of residues from the original RNA sequence is then created. Information about the digestion product’s sequence start or end positions is collected for each residue in the array. Additional information, such as identifying which RNAse was used to generate a particular digestion product and the product length, are also saved. Next, all of the data are compared and each residue in the original RNA sequence gets a status: paired, unpaired or undefined. In the decision stage, the following rules are applied when more than one fragment starts or ends in certain residue and the information provided by fragments is inconsistent: (1) longer fragments have priority, (2) more fragments providing the same paired status information have priority, and (3) when opposite paired status options are represented by the same number of fragments with the same length, the paired status is set to undefined. After the decision stage, the “structure prediction results info page” is sent to user’s web browser. This page will present the original RNA sequence with a paired status for each residue. If the decision is made on the basis of partially inconsistent data, an additional note is shown. The most important part of this page is the form field that contains the constraint commands that can be pasted into the mfold web server for structure visualizations. The user can easily modify the RNAdigest suggested paired status for each residue (constraint commands are automatically actualized). Detailed information for each RNAse is provided at the bottom of the “structure prediction results page”.

### Output

**Figure 4:**
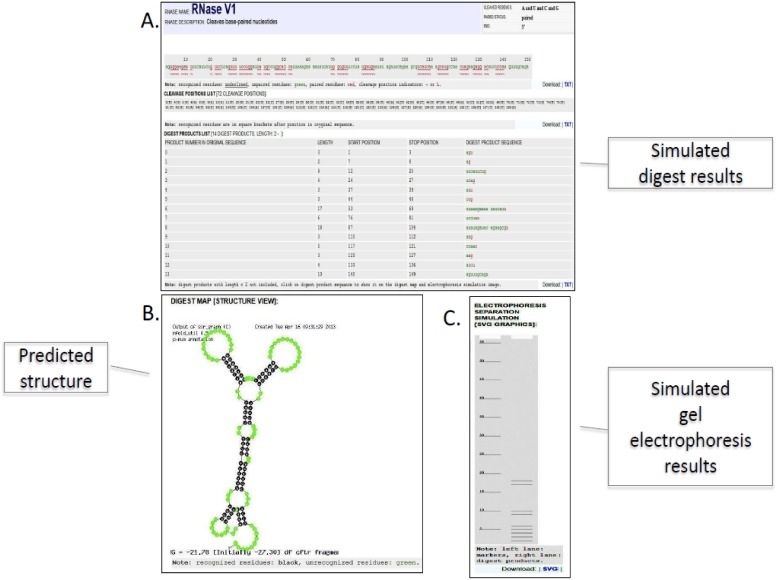
An example of the “digest simulation” mode results screen. **A**. Simulated digestion results are shown. **B**. The predicted RNA structure is shown. **C**. Simulated gel electrophoresis results are shown.

The results of the **“digest simulation” mode** (*Figure 4*) are reported as dynamic tables and images with a color- annotated sequences (paired and unpaired residues, RNAse digestion sites). The tables summarize the cleaved residues, the number of resulting fragments, and positions of cleavage sites. Each chosen RNAse digestion pattern is depicted on a schematic sequencing gel. Graphic representations of resulting digestion fragments on a mfold structure model are also included.

**Figure 5:**
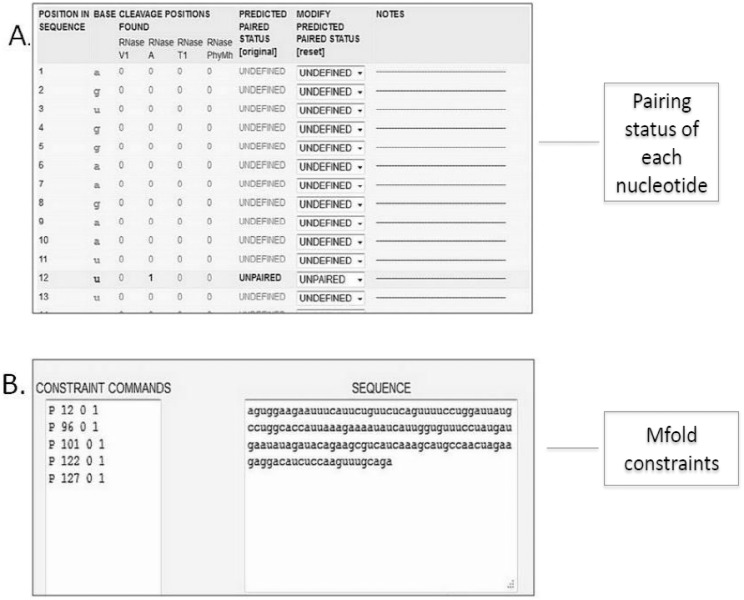
An example “structure prediction” mode results screen. A. The pairing status of each nucleotide is given as an example. B. The mfold constraints are illustrated.

The results of the “**structure prediction**” mode are displayed as input constrains to allow the experimental data to be incorporated into the structural model (*Figure 5*). The residues pairing-status based on experimental data are organized into a dynamic table. This pairing status can be modified by user. All of the data are available to the user as a downloadable text and graphic files.

### Limitations

The accuracy of predicting the cleavage products and sites is limited by the efficiency of the digestion reaction conditions. In our model, the software assumes that the chosen RNAses have equal access to all of the recognized structural motifs, and no additional digestion sites are appearing due to enzymatic structure fragmentation (fragments resulting in digestion of initial structure are not further RNAse substrates). In the case of structural predictions based on RNAse digestion product sequences, the software limits the maximum length of sequence analyses to 200 nt. Fragments shorter than 2 nt are omitted from these analyses.

## Application Examples

**Figure 6:**
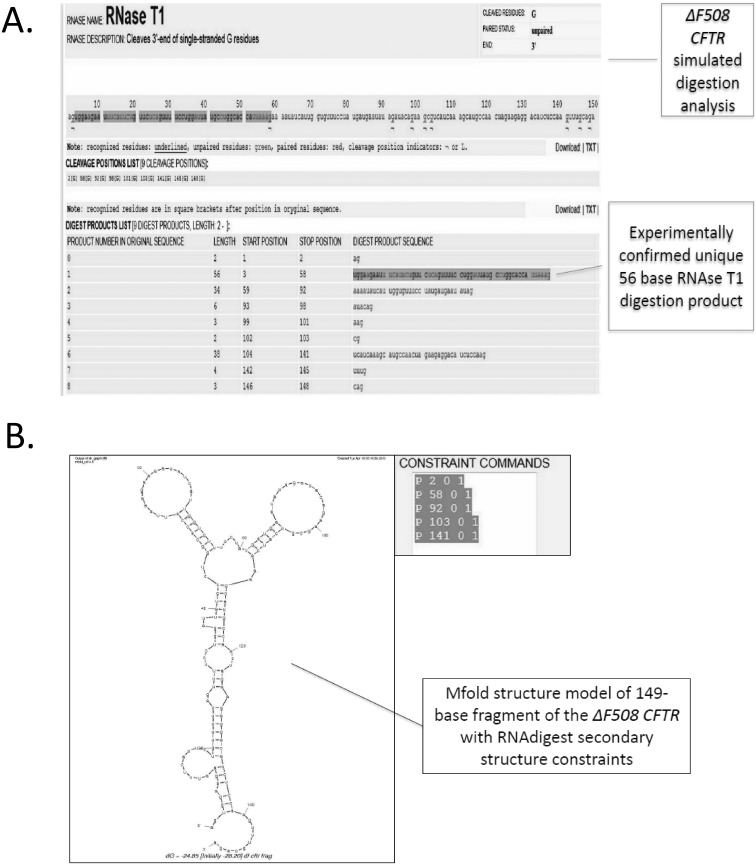
Analyses of the ΔF508 CFTR fragment with RNAdigest RNAse T1 digestion. **A**. The simulated digestion analysis of ΔF508 CFTR is shown that includes a 56-base RNAse T1 digestion product along with other digestion products. **B**. The mfold structure model of the 149-base fragment of ΔF508 CFTR that illustrates the RNAdigest secondary structure constraints.

To validate RNAdigest, we examined the RNAse digestion results of 35 mRNAs and in 33 of those RNAs, the average success rate for predicting the restriction sites was 76% (Table 1). In 2 RNAs, the success rate was only 33% (**Table 1**) [11]. In this latter case, the authors reported that there was some disagreement between their biochemical data and one of their predicted structure models. In *kgmB* mRNA, only 3 out of 36 experimentally detected sites were confirmed via RNA digest, while for *sgm* mRNA the software successfully confirmed 22 out of 39 restriction sites, resulting in 56.4% success rate [11]. Furthermore, RNAdigest successfully confirmed RNA structures using the constraints based on digestion patterns of previously published biochemical data (Figure 6 and Figure S1–S4).

**Table 1:** Summary of RNAdigest analysis on published mRNA structures based on RNAse digestion results*.

Number of mRNAstructures tested	RNAse used	Detected experimentally uniquerestriction sites (total number)	Restriction sites confirmedvia RNAdigest	Reference
2	V1,T1,A, CL3	***75***	***25***	[11]
31	T2, CVR	***100***	***80***	[13]
1	V1,T1,S1	***27***	***21***	[14]
1	T1,T2,A, U2	***21***	***15***	[15]

*Furthermore, RNAdigest correctly identified the presence and position of the 56-base product of RNAse T1 digestion of deltaF508 CFTR fragment (*Figure 6* *,* and *Figures S1–S4*) [16].

Thus, it has to be stressed that RNAdigest predictions are as accurate as the analyzed *in silico* structural models. Our average relative accuracy for RNA secondary structure minimum free energy predictions is 68.6% [12], while the RNAdigest experimental accuracy (including these two less successful mRNA predictions [11]) is 63.2%.

**These Results Validate the Rnadigest Program and Suggest That It Is a Reliable Tool for Designing and Validating the Biochemical Assessment of RNA Structure.**

## Conclusions

The RNAdigest web application offers investigators a valuable tool for the prediction and analyses of enzymatic probes used to determine RNA secondary structure, and allows for fast and reliable experimental results comparison using theoretical structural models. RNAdigest rapidly identifies RNAse cleavage sites, and identifies the sequences and positions of unique structural-related digestion products. The ability to predict the location and sequence of RNAse structure-related digestion products facilitates the design of PCR primers and northern blot probes in order to confirm predicted structural motifs. Thus, these types of analyses make the enzymatic probing of RNA secondary structure easy to adapt for a number of different types of applications.

**Availability and implementation:** Freely available at http://www.biology.pl/rna_digest.

## Supporting Information

Figure S1 Comparative analyses of RNA secondary structures of the WT CFTR (left panel) and ΔF508 CFTR (right panel) fragments with RNAdigest [16]. **A.** Predicted structures models (mfold). **B.** RNAdigest generated graphic stimulation of RNase restriction site positions in the structure models (RNAse recognized residues are shown in black and unrecognized residues are shown in green).
(TIF)

Figure S2 Comparative analyses of RNA secondary structures of the WT CFTR (left panel) and ΔF508 CFTR (right panel) fragments with RNAdigest [16]. **A**. Simulated gel electrophoresis results. **B**. Schematic representation of the location of the digestion products in the RNA sequence (RNAse digestion products are shown are shown in green, products shorter then 2 nt are shown in black).
(TIF)

Figure S3 Comparative analyses of RNA secondary structures of the WT CFTR (left panel) and ΔF508 CFTR (right panel) fragments with RNAdigest [16]. Detailed analysis of these structure models are shown with RNAse A and RNAse V1 digestion and the resulting products sequences are indicated.
(TIF)

Figure S4 Comparative analyses of RNA secondary structures of the WT CFTR (left panel) and ΔF508 CFTR (right panel) fragments with RNAdigest [16]. Detailed analysis of these structure models are shown with RNAse T1 and RNAse PhyMh digestion and resulting products sequences are indicated.
(TIF)
